# The potential and realized foraging movements of bees are differentially determined by body size and sociality

**DOI:** 10.1002/ecy.3809

**Published:** 2022-09-01

**Authors:** Liam K. Kendall, John M. Mola, Zachary M. Portman, Daniel P. Cariveau, Henrik G. Smith, Ignasi Bartomeus

**Affiliations:** ^1^ Centre for Environmental and Climate Science Lund University Lund Sweden; ^2^ U.S. Geological Survey, Fort Collins Science Center Fort Collins Colorado USA; ^3^ Department of Entomology University of Minnesota St. Paul Minnesota USA; ^4^ Department of Biology Lund University Lund Sweden; ^5^ Estación Biológica de Doñana (EBD‐CSIC) Sevilla Spain

**Keywords:** allometry, Anthophila, central place foraging, movement ecology, pollinator conservation

## Abstract

Reversing biodiversity declines requires a better understanding of organismal mobility, as movement processes dictate the scale at which species interact with the environment. Previous studies have demonstrated that species foraging ranges, and therefore, habitat use increases with body size. Yet, foraging ranges are also affected by other life‐history traits, such as sociality, which influence the need of and ability to detect resources. We evaluated the effect of body size and sociality on potential and realized foraging ranges using a compiled dataset of 383 measurements for 81 bee species. Potential ranges were larger than realized ranges and increased more steeply with body size. Highly eusocial species had larger realized foraging ranges than primitively eusocial or solitary taxa. We contend that potential ranges describe species movement capabilities, whereas realized ranges depict how foraging movements result from interactions between species traits and environmental conditions. Furthermore, the complex communication strategies and large colony sizes in highly eusocial species may facilitate foraging over wider areas in response to resource depletion. Our findings should contribute to a greater understanding of landscape ecology and conservation, as traits that influence movement mediate species vulnerability to habitat loss and fragmentation.

## INTRODUCTION

Habitat loss and fragmentation due to anthropogenic land‐use change are leading causes of biodiversity decline (Chase et al., 2020). Mitigating and reversing biodiversity declines requires a better understanding of organismal mobility, as movement processes dictate the scale at which species interact with the environment (Schlägel et al., 2020). Interspecific differences in vulnerability to habitat loss are scale dependent for diverse taxa (e.g., mammals [Tucker et al., 2014], and birds [Hartfelder et al., 2020]), as species differ in life‐history traits that influence movement and connectivity between patches of suitable habitat. Among bees, foraging ranges are positively related to body size (Greenleaf et al., 2007). Therefore, larger bees can exploit resources over more distantly separated habitat patches, reducing their sensitivity to habitat loss (Jauker et al., 2013). However, other life‐history traits, such as sociality, can have profound effects upon animal foraging patterns (Grove, 2012; Jovani et al., 2016; Steffan‐Dewenter & Kuhn, 2003). Yet, little information is known about how body size and sociality jointly influence animal movement patterns, including within well‐studied taxa such as bees.

Interspecific differences in movement processes (e.g., dispersal, migration, and foraging) are structured by species' life‐history traits and how these traits interact with the environment (Schlägel et al., 2020). For central place foragers, such as bees, foraging ranges result from the combined influence of metabolic cost, flight speed, and load size capacity, all of which co‐vary positively with body size (Casey et al., 1985; Everaars et al., 2018; Müller et al., 2006). Yet, the spatial and temporal distribution of resources also plays a central role in animal movement patterns. For example, animals will forage over wider areas in response to low resource availability (e.g., butterflies: Evans et al., 2020; flies: Lander et al., 2011, honeybees: Steffan‐Dewenter & Kuhn, 2003). However, the causes and consequences of changes in resource availability can also be influenced by specific life‐history traits, such as sociality.

Across many animal groups, it is known that social colonies will exploit resources over wider spatial scales, relative to solitary species, because of increased intraspecific competition for local resources (Grove, 2012; Jovani et al., 2016). Ashmole (1963) hypothesized that because social colonies will first deplete nearby resources, this will create a “halo” of low food availability, necessitating wider foraging ranges. Such an effect is then magnified with increasing colony sizes due to increased resource demand (e.g., in seabirds, Jovani et al., 2016). However, such effects have not been tested in other social animal groups. Highly eusocial bee colonies are supergeneralist foragers, with colonies whose densities far exceed those of solitary and primitively eusocial bees (Michener, 1974). Therefore, highly eusocial bee species may induce halo effects, resulting in larger foraging ranges.

Resource exploitation over wider spatial scales by highly eusocial bees may also be facilitated through recruitment communication, whereby foragers or scouts recruit nestmates to high‐quality resource areas (Nieh, 2004; Ratnieks & Shackleton, 2015). For honeybees (*Apis mellifera*), this task division has been hypothesized to explain their larger foraging ranges compared with noncommunicating species, as the relative expenditure of scouting, in terms of energy and risk, can translate into large rewards to the colony through recruitment (Ratnieks & Shackleton, 2015). Yet, this hypothesis remains to be tested beyond honeybees. Therefore, assessing how sociality affects foraging ranges over a wide range of bee species, which differ in their ability to communicate, will deepen our understanding of the origins and benefits of social behaviors.

Differences in the methodology used to measure and define foraging range have important implications for the assessment of ecological processes related to bee movement (please refer to Table 1 for definitions). Previous definitions of bee foraging range have been developed based on species' homing ability (i.e., the proportion of individuals that return to their nest at increasing distances), such that “typical” and “maximum” ranges have been defined at the distance where 50% and 90% released individuals return (cf. Gathmann & Tscharntke, 2002; Greenleaf et al., 2007). However, these measurements more closely approximate potential movement, that is, the distance an individual can move physiologically (or is made to move), without regard for external factors, rather than a measurement of realized movement, that is, the distance individuals travel constrained by external factors (i.e., the real‐world distance). As a result, measurements of potential range are expected to be considerably larger than realized ranges. For example, the homing range of *Bombus terrestris* has been estimated at 9.8 km (Goulson & Stout, 2001), more than double their largest known realized range (3.9 km, Chapman et al., 2003). Therefore, bees most often forage far below proposed economic limits suggested by potential movement, as optimal foraging will not result from foraging at a species' physiological limits, even when resources are scarce (Cresswell et al., 2000). Current models have only used measurements of potential range to predict foraging range (Greenleaf et al., 2007), despite realized estimates being more useful for conservation planning and assessing the scale dependency of ecosystem functioning associated with bee foraging patterns.

**TABLE 1:** Definitions of foraging range and the methods used to measure it.

Metric	Definition
Potential movement^a^	
Feeder	In feeder‐training experiments, workers of highly eusocial species are trained to forage on an artificial food source, which is then gradually moved further away from the colony location, until workers no longer forage upon the resource. The distance at which foraging ceases is used to estimate maximum foraging range. Using this method, only maximum ranges can be measured as measurements are directly related to the artificial food source, rather than the spatial distribution of resources surrounding the nest.
Homing	In homing studies, bees are taken from their nest, marked, and released at increasing distances from their nests. This method has commonly been used to estimate typical and maximum foraging range using logistic regression to estimate the distance at which 50% and 90% of foragers are lost. Some studies report less than 50% of foragers returning at the closest distances. For these studies, only a maximum foraging range can be calculated. Nine studies did not provide enough information to calculate 50% and 90% loss rates. For these studies, we used the highest observed distance at which a bee returned as an estimate of maximum foraging range.
Realized movement^b^	
Associative	Associative studies measure foraging range by associating known nests, colonies, and bee populations and known floral resources, such as managed hives and the observed distance of hive activity within a crop. For these studies, only a maximum foraging range could be estimated, which we defined as the largest recorded associative distance between bees and floral resources or, for one study, the spatial scale at which each the density of each species responded to the availability of mass‐flowering resources (Westphal et al., 2006).
Molecular	Molecular methods are widely used to estimate foraging ranges in eusocial species. Most commonly, foragers are collected throughout the landscape at different locations. These foragers are then genotyped, from which genetic markers are used to identify and assign foragers to colonies based upon sibship analyses. The distance between full siblings is then used to approximate foraging range. For these studies, typical foraging ranges were the average distance between siblings and colonies. Maximum foraging ranges were the largest observed distance.
Tracking	These studies involve placing transmitters on individual bees, from which location information during foraging bouts can be recorded. For these studies, we estimated typical foraging range as the average distance across tracked flights and maximum foraging range as the largest observed distance during any flight.
Mark‐recapture	In these studies, foraging bees are captured and marked, such as with apiarist tags, upon leaving their nests. Marked individuals are then searched for in the surrounding environment to estimate foraging range. For these studies, we estimated typical foraging range as the average distance traveled among recaptured bees and maximum foraging range as the largest observed distance traveled by any recaptured bee.
Waggle dance	These studies directly measured waggle dance communication within honeybees (*Apis* spp.). Returning individuals communicate distance and direction of floral resources using waggle dances, are then decoded to estimate foraging range. For these studies, we estimated typical foraging range as the average distance among decoded waggle dances, and maximum foraging range as the largest observed waggle dance distance observed within the study.

^a^ Methods that estimate the distance bees move under physiological constraints (or is made to move), with limited regard for external factors. These estimates reflect the fundamental or physiological limits within which a bee can forage without being lost.

^b^ Methods that estimate the distance bees move when constrained by external factors (i.e., real‐world distance). These estimates reflect actual foraging ranges affected by local and landscape factors such as food availability and environmental conditions.

In this study, we assess how two types of bee foraging range (potential or realized movement) are structured by interspecific differences in sociality and body size. Specifically, we assess, (i) how potential and realized bee foraging ranges scale with body size, (ii) how foraging range differs among bee species with differing degrees of sociality, and (iii) how our model predictions improve upon pre‐existing models.

## MATERIALS AND METHODS

To identify relevant publications for our study, we first compiled measurements of potential and realized foraging range from previous reviews (Greenleaf et al., 2007; Zurbuchen et al., 2010). We then undertook a systematic Scopus database search (title, abstracts, and keywords) with the following search terms: (bee OR apoid*) AND (forag* AND distanc*) OR (“flight range” OR “foraging range”). As of 13 January 2022, this returned 738 articles. We then screened all articles published after Greenleaf et al. (2007) for foraging range measurements. We focused solely on female bee foraging patterns, so excluded the few studies of dispersal. From our search, in combination with both previous review databases (Greenleaf et al., 2007; Zurbuchen et al., 2010), we compiled measurements of foraging range from 91 publications (Appendix S1: Section S1). For each measurement, we defined their method of calculation and the type of range (potential typical, potential maximum, realized typical, realized maximum) (please refer to Table 1). Our collation resulted in 61 new measurements of homing range, and 11 new feeder‐training measurements, relative to those used in the models by Greenleaf et al. (2007), as well as 306 additional new measurements. We also compiled the reported sample sizes of each study where available (Appendix S2). We found 42 additional measurements (from 18 publications) that could not be classified as either a typical or maximum range. These were not included in our analyses but are included in the Appendix for general interest (Appendix S1: Section S2).

We checked the validity of species' names following (Ascher & Pickering, 2020). We assigned female body size measurements, that is, the intertegular distance (ITD: Kendall et al., 2019) and degree of sociality to each species from literature sources (Appendix S1: Section S3). Across social species, all measurements were taken from workers. If we found multiple ITD measurements for a given species, we used the mean value. We then converted ITD values to body mass (mg), using a published predictive model for bees (Kendall et al., 2019). We characterized species into three levels of sociality: obligate solitary, primitively eusocial, or highly eusocial. Definitions of sociality follow Michener (2007). Obligate solitary species are those bee species that provision their own nest cells. This group includes species that live communally or in aggregations. Primitively eusocial species are those species that have nonpermanent colonies, generally founded by a single female. This group included bumblebees (*Bombus* spp.), *Lasioglossum umbripenne*, and *Xylocopa virginica*. As it can be difficult to lump primitively eusocial species into a single group, we also considered an alternate model in which *Xylocopa virginica* was considered solitary, which led to near‐identical results. Highly eusocial species are those species that have permanent colonies, which reproduce through groups or swarms. This group included honeybees (*Apis* species), and stingless bees (*Meliponini*).

All statistical analyses were undertaken in R (v.4.1.0, R Core Team, 2021). We fitted Bayesian generalized linear mixed models using the *brms* package (v2.16.3, Bürkner, 2017) to analyze how bee foraging ranges related to range type (categorical: four levels), body size (mass, mg) and eusociality (categorical: three levels). For these purposes, we specified two models: (1) Foraging range ~ body size × range type, and (2) Foraging range ~ body size × range type × eusociality. As allometric relationships typically follow a power function, we log‐transformed both body size and foraging range prior to analyses, which improved fit (Kendall et al., 2019). In both models, the random effect structure consisted of varying intercepts for: (1) publication, (2) metric type, (3) a phylogenetic effect between bee genera, and (4) a nonphylogenetic species‐level effect (Nakagawa & Santos, 2012). For the phylogenetic effect, we constructed a phylogenetic correlation matrix using an available bee genera tree (Hedtke et al., 2013), from which we pruned nonrepresented genera and fitted a chronogram by penalized likelihood using the *ape* package (v.5.5, Paradis & Schliep, 2019). We compared the predictive accuracy of our models by calculating and comparing the marginal (*R* ^2^_mar_) and conditional (*R* ^2^_con_) *R* ^*2*^ using the *performance* package (v.0.7.2, Lüdecke et al., 2021).

We parameterized each model with a Gaussian distribution, which was run for 3000 iterations (burn‐in of 1500 iterations). We set weakly informative priors and manipulated Δ and maximum tree depth to avoid divergent transitions. Model parameters, posterior predictive checks, and assessments of chain convergence are available from the provided R code. A full evaluation of the predictive accuracy of our best‐fitting model is provided in Appendix S3.

## RESULTS

**FIGURE 1:**
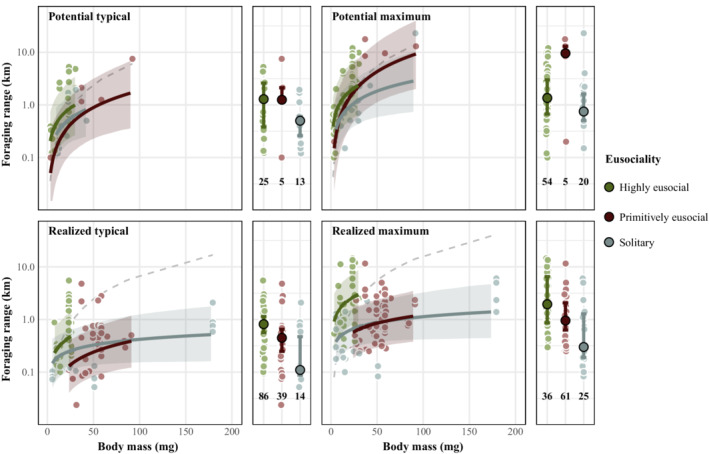
Posterior estimates of potential (upper) and realized (lower) estimates of foraging ranges (typical left, maximum right) as a function of body size (body mass, mg) and degree of sociality. Bold lines and ribbons indicate lines of best fit and corresponding 95% credible intervals. The median, interquartile range, and sample size for each social group are displayed in the smaller panels. Dashed lines indicate model predictions from Greenleaf et al. (2007)’s typical or maximum homing range models. In all panels, background circles denote raw data. *y*‐axis is shown on a log_10_ scale.

In total, we collated 383 measurements of foraging range: potential foraging range, *n* = 122 (maximum = 79, typical = 43), and realized foraging range, *n* = 261 (maximum: *n* = 122, typical: *n* = 139). Foraging range measurements came from six families, 29 genera and 81 species (Appendix S2). Honeybees (131 measurements, 97 from *A. mellifera*) and bumblebees (106 measurements, 32 from *B. terrestris*) were the most studied taxa. Median potential foraging ranges were 1.29 km (typical) and 1.35 km (maximum) for highly eusocial species, 1.25 km (typical) and 9.58 km (maximum) for primitively eusocial species, and 500 m (typical) and 750 m (maximum) for solitary bees (Figure 1). Median realized foraging ranges were 816 m (typical) and 1.95 km (maximum) for highly eusocial species, 448 m (typical) and 955 m for primitively eusocial species, and 110 m (typical) and 300 m (maximum) for solitary bees.

We found that potential and realized foraging ranges scaled positively with body size (*R* ^2^_mar_: 0.36, *R* ^2^_con_: 0.82; Figure 1). Potential ranges were larger than realized foraging ranges and increased at a greater rate per unit body size. Including eusociality in our model markedly increased model predictive accuracy (*R* ^2^_mar_: 0.43, *R* ^2^_con_: 0.83). Highly eusocial bees exhibited greater foraging ranges than either primitively eusocial or solitary species, when controlling for body size. In contrast, primitively eusocial and solitary species exhibited highly similar foraging range estimates.

Differences among social groups were most pronounced for realized maximum range. For three equal‐sized bee species that differ in their degree of sociality (i.e., body mass: 23 mg, the size of a honeybee), the highly eusocial bee's realized foraging range was 357% or 2 km larger than the primitively eusocial bee, and 294% or 1.92 km larger than the solitary bee (please refer to Appendix S3 for further analyses).

## DISCUSSION

We assessed how the potential and realized foraging ranges of bees were related to interspecific differences in body size and sociality. We found that potential foraging ranges were considerably larger than realized ranges, scaled steeply with body size, but varied only marginally despite differences in sociality. This demonstrates that, in the absence of external limiting factors, body size is a key trait determining the physiological limits over which animals, such as bees can move. Critically, realized ranges scaled trivially with body size but varied largely due to sociality, most notably for maximum ranges. As such, realized ranges are more reflective of how animal movements result from interactions between environmental factors (e.g., resource availability) and species' life‐history traits that dictate movement capabilities (Schlägel et al., 2020). Animals will alter their movement patterns in response to changes in resource availability, for example, by increasing their foraging or dispersal range in resource‐poor habitats (e.g., in butterflies: Evans et al., 2020, and flies: Lander et al., 2011). Similar patterns also occur in bees, irrespective of sociality (e.g., honeybees: Steffan‐Dewenter & Kuhn, 2003, bumblebees: Jha & Kremen, 2013, and solitary bees: Minckley et al., 1994). Therefore, our results contend that ecological processes related to colonial living and social behaviors, which affect both resource availability and choice, result in wider foraging ranges among highly eusocial species than other taxa.

Social animal colonies can have a large effect on local resource availability, in which large population densities deplete available resources. This increases resource competition among colony members, inducing wider foraging ranges, as is seen in colony‐nesting seabirds (Jovani et al., 2016), as well as group foraging primates (Grove, 2012). Our results suggest that similar patterns in local resource depletion may result in wider foraging ranges for highly eusocial bees. For example, honeybee colonies disproportionately deplete floral resources, relative to solitary bees (Cane & Tepedino, 2017), which can lead to local resource scarcity (Thomson, 2004). Furthermore, honeybee foraging ranges increase in response to colony growth (Schneider & McNally, 1993) and reduced resource availability (Steffan‐Dewenter & Kuhn, 2003). The same mechanism can be expected to apply to stingless bees. Veiga et al. (2013) showed that *Melipona* colonies produced larger workers in response to increased colony food stores, which may suggest that increased resource collection necessitates foraging over wider areas. Larger foraging ranges may then be further facilitated by communicative behaviors (Nieh, 2004; Ratnieks & Shackleton, 2015). Communication in insect societies improves colony‐level foraging efficiency through increased recruitment to high‐quality resources, which then lessens the costs and risks of foraging over wider areas (Czaczkes et al., 2015; Ratnieks & Shackleton, 2015). As a result, wider foraging ranges among highly eusocial taxa represent a cost‐effective strategy to overcome resource competition and/or resource‐poor environments.

We found that primitively eusocial and solitary bees had highly similar realized foraging ranges. Short foraging ranges among these taxa are likely to result from their occurrence being closely tied to floral resource availability during critical life‐history stages, such as nest establishment. Both solitary bees, as well as nest‐searching bumblebee queens, can exhibit preferences for nesting sites near floral resources (Julier & Roulston, 2009; Suzuki et al., 2009). Solitary bees can also actively move their nest location in response to reduced resource availability, for example, after disturbance events (Franzén et al., 2009). Furthermore, Gathmann and Tscharntke (2002) found no differences between the foraging ranges of polylectic and oligolectic solitary bee species. Therefore, although foraging ranges in these species do vary in relation to resource availability (Jha & Kremen, 2013; Minckley et al., 1994), their close association with floral resources during nest establishment, as well as their lower population densities compared with highly eusocial species (Michener, 1974), suggest that variability in foraging ranges is more likely to result from phenological changes in resource availability, rather than responses to local resource depletion with increasing distance from nesting locations.

Trait‐based approaches to assessing animal movement are central to the broader understanding of landscape ecology and conservation. Human‐dominated landscapes are characterized by habitat fragmentation, and animal movements, including foraging and dispersal, are crucial for the persistence of biodiversity in such landscapes because they connect resources, genes, and processes between separated ecological communities and ecosystems (Schlägel et al., 2020). The positive relationship between body size and foraging range is well established across animal taxa (birds: Hartfelder et al., 2020; mammals: Tucker et al., 2014). As a result, larger species can exhibit greater connectivity between habitat patches, reducing their vulnerability to future habitat loss (Hartfelder et al., 2020). However, our results highlight that life‐history traits related to resource collection, such as sociality, play a key role in structuring range sizes, in accordance with studies in other taxa (e.g., diet in mammals; Tucker et al., 2014). As a result, among bees, small‐bodied primitively eusocial and solitary taxa are likely to be most vulnerable to habitat loss and fragmentation, although assessments should also consider other known causes of decline (e.g., specialization, Goulson et al., 2008). Importantly, pre‐existing estimates of bee foraging ranges based upon potential movement (Greenleaf et al., 2007) may have overestimated realized foraging ranges (please refer to Appendix S3), welcoming reassessments of the scale dependency of habitat conservation assessments and ecosystem services (e.g., Kennedy et al., 2013). We provide our models within the R package *pollimetry* (Kendall et al., 2019) to facilitate further investigation into ecological processes related on bee foraging ranges. In conclusion, our study underscores that trait‐based assessments of animal movement are a crucial component for designing relevant ecological studies, as well as the conservation of both animal populations and communities.

## AUTHOR CONTRIBUTIONS

Liam K. Kendall, John M. Mola, and Ignasi Bartomeus designed the study. Liam K. Kendall and John M. Mola devised and undertook the literature review. Zachary M. Portman, Liam K. Kendall, and John M. Mola extracted all life‐history trait information. Liam K. Kendall, with assistance from Ignasi Bartomeus, undertook all analyses. Liam K. Kendall drafted the manuscript, and all authors contributed substantially to the final version.

## CONFLICT OF INTEREST

The authors declare no conflict of interest.

## Supporting information


Appendix S1

Appendix S2

Appendix S3

## Data Availability

All data, primary references, and R code (Kendall et al., 2022) are available in Figshare at https://doi.org/10.6084/m9.figshare.18857654.
