# Supplementary material for: The potential and realized foraging movements of bees are differentially determined by body size and sociality
Source: Ecology. 2022 Sep 1;103(11):e3809. doi: 10.1002/ecy.3809 (PMC9786665; doi:10.1002/ecy.3809)
Supplement: Supplementary file 1 — Appendix S1 [file ECY-103-e3809-s002.pdf]

Authors: Liam K. Kendall, John M. Mola, Zachary M. Portman, Daniel P. Cariveau, Henrik G. Smith, Ignasi Bartomeus

Title: The potential and realized foraging movements of bees are differentially determined by body size and sociality

Journal: Ecology

Appendix S1. Literature sources for bee foraging range measurements and life-history traits

Section S1. Reference list of all publications that provided foraging range measurements for our synthesis.

1. Abrol, D. P. (1986). Flight range and significance of wing hooks in *Megachile femorata* Smith (Hymenoptera: Megachilidae). *Journal of animal morphology and physiology*, *33*(1-2), 107-112.
2. Abrol, D. P. (1988). Foraging range of subtropical bees, *Megachile flavipes, Megachile nana* (Hymenoptera: Megachilidae) and *Apis florea* (Hymenoptera: Apidae). *Journal of the Indian Institute of Science*, *68*(1–2), 43.
3. Amand, P. C. St., Skinner, D. Z., & Peaden, R. N. (2000). Risk of alfalfa transgene dissemination and scale-dependent effects: *Theoretical and Applied Genetics*, *101*(1–2), 107–114. https://doi.org/10.1007/s001220051457
4. Appanah, S. (1982). Pollination of Androdioecious *Xerospermum intermedium* Radlk. (Sapindaceae) in a rain forest. *Biological Journal of the Linnean Society*, *18*(1), 11–34. https://doi.org/10.1111/j.1095-8312.1982.tb02031.x
5. Araújo, E. D., Costa, M., Chaud-Netto, J., & Fowler, H. G. (2004). Body size and flight distance in stingless bees (Hymenoptera: Meliponini): Inference of flight range and possible ecological implications. *Brazilian Journal of Biology*, *64*, 563–568.
6. Bänsch, S., Tscharntke, T., Ratnieks, F. L., Härtel, S., & Westphal, C. (2020). Foraging of honey bees in agricultural landscapes with changing patterns of flower resources. *Agriculture, Ecosystems & Environment*, *291*, 106792.
7. Beekman, M., & Ratnieks, F. L. W. (2000). Long-range foraging by the honey-bee, *Apis mellifera* L.: *Honey-bee foraging*. *Functional Ecology*, *14*(4), 490–496. https://doi.org/10.1046/j.1365-2435.2000.00443.x
8. Beekman, M., Sumpter, D. J. T., Seraphides, N., & Ratnieks, F. L. W. (2004). Comparing foraging behaviour of small and large honey-bee colonies by decoding waggle dances made by foragers. *Functional Ecology*, *18*(6), 829–835. https://doi.org/10.1111/j.0269-8463.2004.00924.x
9. Campbell, A. J., Gomes, R. L. C., da Silva, K. C., & Contrera, F. A. L. (2019). Temporal variation in homing ability of the neotropical stingless bee *Scaptotrigona aff. postica* (Hymenoptera: Apidae: Meliponini). *Apidologie*, *50*(5), 720–732. https://doi.org/10.1007/s13592-019-00682-z
10. Carvell, C., Jordan, W. C., Bourke, A. F. G., Pickles, R., Redhead, J. W., & Heard, M. S. (2012). Molecular and spatial analyses reveal links between colony-specific foraging distance and landscape-level resource availability in two bumblebee species. *Oikos 121*, 734–742.
11. Cavigliasso, P., Phifer, C. C., Adams, E. M., Flaspohler, D., Gennari, G. P., Licata, J. A., & Chacoff, N. P. (2020). Spatio-temporal dynamics of landscape use by the bumblebee *Bombus pauloensis* (Hymenoptera: Apidae) and its relationship with pollen provisioning. *PloS One*, *15*(7), e0216190.
12. Chapman, R. E., Wang, J., & Bourke, A. F. G. (2003). Genetic analysis of spatial foraging patterns and resource sharing in bumble bee pollinators. *Molecular Ecology*, *12*(10), 2801–2808. https://doi.org/10.1046/j.1365-294X.2003.01957.x
13. Charman, T. G., Sears, J., Green, R. E., & Bourke, A. F. G. (2010). Conservation genetics, foraging distance and nest density of the scarce Great Yellow Bumblebee (*Bombus distinguendus*). *Molecular Ecology*, *19*(13), 2661–2674. https://doi.org/10.1111/j.1365-294X.2010.04697.x
14. Chmurzyński, J. A., Kieruzel, M., Krzysztofiak, A., & Krzysztofiak, L. (2010). Long-distance homing ability in *Dasypoda altercator* (Hymenoptera, Melittidae). *Ethology*, *104*(5), 421–429. https://doi.org/10.1111/j.1439-0310.1998.tb00080.x

15. Connop, S., Hill, T., Steer, J., & Shaw, P. (2010). Microsatellite analysis reveals the spatial dynamics of *Bombus humilis* and *Bombus sylvarum*. *Insect Conservation and Diversity*, *4*. 212-221.
16. Costa, L., Nunes-Silva, P., Galaschi-Teixeira, J. S., Arruda, H., Veiga, J. C., Pessin, G., de Souza, P., & Imperatriz-Fonseca, V. L. (2021). RFID-tagged amazonian stingless bees confirm that landscape configuration and nest re-establishment time affect homing ability. *Insectes Sociaux*, *68*(1), 101–108.
17. Couvillon, M. J., Riddell Pearce, F. C., Accleton, C., Fensome, K. A., Quah, S. K. L., Taylor, E. L., & Ratnieks, F. L. W. (2015). Honey bee foraging distance depends on month and forage type. *Apidologie*, *46*(1), 61–70. https://doi.org/10.1007/s13592-014-0302-5
18. Crowther, L. P., Wright, D. J., Richardson, D. S., Carvell, C., & Bourke, A. F. G. (2019). Spatial ecology of a range-expanding bumble bee pollinator. *Ecology and Evolution*, *9*(3), 986–997. https://doi.org/10.1002/ece3.4722
19. da Silva Correia, F. C., Peruquetti, R. C., & Pires, G. A. (2020). Determinação da área de forrageamento da espécie *Melipona grandis* (Apidae: Meliponina) pelo método de captura e recaptura. *Oecologia Australis*, *24*(3), 714–720.
20. Danner, N., Keller, A., Härtel, S., & Steffan-Dewenter, I. (2017). Honey bee foraging ecology: Season but not landscape diversity shapes the amount and diversity of collected pollen. *PLOS ONE*, *12*(8), e0183716. https://doi.org/10.1371/journal.pone.0183716
21. Danner, N., Molitor, A. M., Schiele, S., Härtel, S., & Steffan-Dewenter, I. (2016). Season and landscape composition affect pollen foraging distances and habitat use of honey bees. *Ecological Applications*, *26*(6), 10.
22. Darvill, B., Knight, M. E., & Goulson, D. (2004). Use of genetic markers to quantify bumblebee foraging range and nest density. *Oikos*, *107*(3), 471–478. https://doi.org/10.1111/j.0030-1299.2004.13510.x
23. Desjardins, E. V.-C., & Oliveira, D. D. (2006). Commercial bumble bee *Bombus impatiens* (Hymenoptera: Apidae) as a pollinator in lowbush blueberry (Ericale: Ericaceae) fields. *Journal of Economic Entomology*, *99*(2), 7.
24. Dhaliwal, H. S., & Sharma, P. L. (1973). The foraging range of the Indian honeybee on two crops. *Journal of Apicultural Research*, *12*(2), 131–134.
25. Dyer, F. C., & Seeley, T. D. (1991). Dance dialects and foraging range in three Asian honey bee species. *Behavioral Ecology and Sociobiology*, *28*(4). https://doi.org/10.1007/BF00175094
26. Esser, J. (2005). *Die Seidenbiene Colletes daviesanus Smith 1846. Lebensstrategie einer spezialisierten Wildbiene*.
27. Franzen, M., Larsson, M., & Nilsson, S. G. (2009). Small local population sizes and high habitat patch fidelity in a specialised solitary bee. *Journal of Insect Conservation*, 7.
28. Frisch, K. von. (1967). *Dance language and orientation of bees*. Harvard University Press.
29. Garbuzov, M., Schürch, R., & Ratnieks, F. L. W. (2015). Eating locally: Dance decoding demonstrates that urban honey bees in Brighton, UK, forage mainly in the surrounding urban area. *Urban Ecosystems*, *18*(2), 411–418. https://doi.org/10.1007/s11252-014-0403-y
30. Gary, N. E., Witherell, P. C., & Marston, J. (1972). Foraging range and distribution of honey bees used for carrot and onion pollination. *Environmental Entomology*, *1*(1), 8.
31. Gathmann, A., & Tscharntke, T. (2002). Foraging ranges of solitary bees. *Journal of Animal Ecology*, *71*(5), 757–764. https://doi.org/10.1046/j.1365-2656.2002.00641.x
32. Geib, J. C., Strange, J. P., & Galen, C. (2015). Bumble bee nest abundance, foraging distance, and host-plant reproduction: Implications for management and conservation. *Ecological Applications*, *25*(3), 768–778. https://doi.org/10.1890/14-0151.1
33. Goulson, D., & Stout, J. C. (2001). Homing ability of the bumblebee *Bombus terrestris* (Hymenoptera: Apidae). *Apidologie*, *32*(1), 105–111. https://doi.org/10.1051/apido:2001115
34. Greenleaf, S. S., Williams, N. M., Winfree, R., & Kremen, C. (2007). Bee foraging ranges and their relationship to body size. *Oecologia*, *153*(3), 589–596. https://doi.org/10.1007/s00442-007-0752-9
35. Guedot, C., Bosch, J., & Kemp, W. P. (2009). Relationship between body size and homing ability in the genus *Osmia* (Hymenoptera; Megachilidae). *Ecological Entomology*, *34*(1), 158–161.
36. Hagen, M., Wikelski, M., & Kissling, W. D. (2011). Space use of bumblebees (*Bombus* spp.) revealed by radio-tracking. *PLoS ONE*, *6*(5), e19997. https://doi.org/10.1371/journal.pone.0019997
37. Hagler, J. R., Mueller, S., Teuber, L. R., Machtley, S. A., & Van Deynze, A. (2011). Foraging range of honey bees, *Apis mellifera*, in alfalfa seed production fields. *Journal of Insect Science*, *11*(144), 1–12. https://doi.org/10.1673/031.011.14401
38. Hembach, J. (1993). *Zur Stechimmenfauna (Hymenoptera, Aculeata) des Landkreises Daun/Eifel*. Universität zu Köln.
39. Herrmann, M. (2000). *Ökologisch-faunistische Untersuchungen an Bienen und Wespen in einer extensiv genutzten Agrarlandschaft (Hymenoptera, Aculeata)*. Cuvillier Verlag.

40. Hofmann, M. M., Fleischmann, A., & Renner, S. S. (2020). Foraging distances in six species of solitary bees with body lengths of 6 to 15 mm, inferred from individual tagging, suggest 150 m-rule-of-thumb for flower strip distances. *Journal of Hymenoptera Research*, *77*, 105–117. https://doi.org/10.3897/jhr.77.51182
41. Jaffé, R., Castilla, A., Pope, N., Imperatriz-Fonseca, V. L., Metzger, J. P., Arias, M. C., & Jha, S. (2016). Landscape genetics of a tropical rescue pollinator. *Conservation Genetics*, *17*(2), 267–278. https://doi.org/10.1007/s10592-015-0779-0
42. Janzen, D. H. (1971). Euglossine bees as long-distance pollinators of tropical plants. *Science*, *171*(3967), 203–205. https://doi.org/10.1126/science.171.3967.203
43. Jha, S., & Kremen, C. (2013). Resource diversity and landscape-level homogeneity drive native bee foraging. *Proceedings of the National Academy of Sciences*, *110*(2), 555–558. https://doi.org/10.1073/pnas.1208682110
44. Kaehler, T. G., Halinski, R., Contrera, F. A., Silveira, A., & Blochtein, B. (2021). Flight distance and foraging of *Tetragonisca fiebrigi* (Apidae: Meliponini) in response to different concentrations of sugar in food resources and abiotic factors. *Journal of Apicultural Research*, 1–13.
45. Kitamura, T., & Maeta, Y. (1969). Studies on the pollination of apple by *Osmia*. III. Preliminary report on the homing ability of *Osmia cornifrons* (Radoszkowski) and *O. pedicornis* Cockerell. *Kontyu*, *37*(1), 83–90.
46. Knight, M. E., Martin, A. P., Bishop, S., Osborne, J. L., Hale, R. J., Sanderson, R. A., & Goulson, D. (2005). An interspecific comparison of foraging range and nest density of four bumblebee (*Bombus*) species. *Molecular Ecology*, *14*(6), 1811–1820. https://doi.org/10.1111/j.1365-294X.2005.02540.x
47. Kohl, P. L., Thulasi, N., Rutschmann, B., George, E. A., Steffan-Dewenter, I., & Brockmann, A. (2020). Adaptive evolution of honeybee dance dialects. *Proceedings of the Royal Society B: Biological Sciences*, *287*(1922), 20200190. https://doi.org/10.1098/rspb.2020.0190
48. Kreyer, D., Oed, A., Walther-Hellwig, K., & Frankl, R. (2004). Are forests potential landscape barriers for foraging bumblebees? Landscape scale experiments with *Bombus terrestris* agg. and *Bombus pascuorum* (Hymenoptera, Apidae). *Biological Conservation*, 8.
49. Kuhn-Neto, B., Contrera, F. A. L., Castro, M. S., & Nieh, J. C. (2009). Long distance foraging and recruitment by a stingless bee, *Melipona mandacaia*. *Apidologie*, *40*(4), 472–480. https://doi.org/10.1051/apido/2009007
50. Layek, U., Bisui, S., & Karmakar, P. (2021). Flight range and resource loading-unloading behavior of stingless bee *Tetragonula iridipennis* (Smith). *Journal of Apicultural Research*, 1–12.
51. Michener, C. D. (1974). *The social behavior of the bees: A comparative study*. Harvard University Press.
52. Mola, J. M., Miller, M. R., O'Rourke, S. M., & Williams, N. M. (2020). Forests do not limit bumble bee foraging movements in a montane meadow complex. *Ecological Entomology*, *45*(5), 955–965. https://doi.org/10.1111/een.12868
53. Molitor, A. (1937). Zur vergleichenden Psychobiologie der akuleaten Hymenopteran auf experimenteller Grundlage. *Biologica Generalis*, *13*, 294–333.
54. Münster-Swendsen, M. (1968). On the biology of the solitary bee *Panurgus banksianus* Kirby (Hymenoptera, Apidae), including some ecological aspects. *Arsskrift Kongelige Veterinaer Og Landbohojskole*, *51*, 215–241.
55. Nagamitsu, T., Tsukuba, S., Ushirokita, F., & Konno, Y. (2012). Foraging habitats and floral resource use by colonies of long-and short-tongued bumble bee species in an agricultural landscape with kabocha squash fields. *Applied Entomology and Zoology*, *47*(3), 181–190.
56. Nagamitsu, T., & Yamagishi, H. (2009). Nest density, genetic structure, and triploid workers in exotic *Bombus terrestris* populations colonized Japan. *Apidologie*, *40*(4), 429–440.
57. Neff, J. L., & Danforth, B. N. (1991). The nesting and foraging behavior of *Perdita texana* (Cresson) (Hymenoptera: Andrenidae). *Journal of the Kansas Entomological Society*, *64*(4), 394–405.
58. Nunes-Silva, P., Costa, L., Campbell, A. J., Arruda, H., Contrera, F. A. L., Teixeira, J. S. G., Gomes, R. L. C., Pessin, G., Pereira, D. S., de Souza, P., & Imperatriz-Fonseca, V. L. (2020). Radiofrequency identification (RFID) reveals long-distance flight and homing abilities of the stingless bee *Melipona fasciculata*. *Apidologie*, *51*(2), 240–253. https://doi.org/10.1007/s13592-019-00706-8
59. Osborne, J. L., Clark, S. J., Morris, R. J., Williams, I. H., Riley, J. R., Smith, A. D., Reynolds, D. R., & Edwards, A. S. (1999). A landscape-scale study of bumble bee foraging range and constancy, using harmonic radar. *Journal of Applied Ecology*, *36*(4), 519–533. https://doi.org/10.1046/j.1365-2664.1999.00428.x

60. Osborne, J. L., Martin, A. P., Carreck, N. L., Swain, J. L., Knight, M. E., Goulson, D., Hale, R. J., & Sanderson, R. A. (2008). Bumblebee flight distances in relation to the forage landscape. *Journal of Animal Ecology*, *77*(2), 406–415. https://doi.org/10.1111/j.1365-2656.2007.01333.x
61. Osborne, J. L., Smith, A., Clark, S. J., Reynolds, D. R., Barron, M. C., Lim, K. S., & Reynolds, A. M. (2013). The ontogeny of bumblebee flight trajectories: From naive explorers to experienced foragers. *PLoS ONE*, *8*(11), 11.
62. Packer, J. S. (1970). *The flight and foraging behavior of the alkali bee (Nomia melanderi) and the alfalfa leaf-cutter bee (Megachile rotundata)*. Utah State University.
63. Pahl, M., & Zhu, H. (2011). Large scale homing in honeybees. *PLoS ONE*, *6*(5), 7.
64. Pasquet, R. S., Peltier, A., Hufford, M. B., Oudin, E., Saulnier, J., Paul, L., Knudsen, J. T., Herren, H. R., & Gepts, P. (2008). Long-distance pollen flow assessment through evaluation of pollinator foraging range suggests transgene escape distances. *Proceedings of the National Academy of Sciences*, *105*(36), 13456–13461. https://doi.org/10.1073/pnas.0806040105
65. Rao, S., Hoffman, G., Kirby, J., & Horne, D. (2019). Remarkable long-distance returns to a forage patch by artificially displaced wild bumble bees (Hymenoptera: Apidae). *Journal of Apicultural Research*, *58*(4), 522–530. https://doi.org/10.1080/00218839.2019.1584962
66. Rao, S., & Strange, J. P. (2012). Bumble bee (Hymenoptera: Apidae) foraging distance and colony density associated with a late-season mass flowering crop. *Environmental Entomology*, *41*(4), 11.
67. Rau, P. (1929). Experimental studies in the homing of carpenter and mining bees. *Journal of Comparative Psychology*, *9*(1), 35–70. https://doi.org/10.1037/h0076024
68. Rau, P. (1931). Additional experiments on the homing of carpenter- and mining-bees. *Journal of Comparative Psychology*, *12*(3), 257–261. https://doi.org/10.1037/h0072864
69. Redhead, J. W., Dreier, S., Bourke, A. F. G., Heard, M. S., Jordan, W. C., Sumner, S., Wang, J., & Carvell, C. (2016). Effects of habitat composition and landscape structure on worker foraging distances of five bumble bee species. *Ecological Applications*, *26*(3), 726–739. https://doi.org/10.1890/15-0546
70. Rodrigues, F., & Ribeiro, M. D. F. (2014). How far can *Melipona mandacaia* (Hymenoptera, Apidae, Meliponini) foragers fly in a caatinga area of NE brazil? *Sociobiology*, *61*(4), 523–528. https://doi.org/10.13102/sociobiology.v61i4.523-528
71. Roubik, D. W., & Aluja, M. (1983). Flight ranges of *Melipona* and *Trigona* in tropical forest. *Journal of the Kansas Entomological Society*, 217–222.
72. Saville, N. (1997). Bumblebee movement in a fragmented agricultural landscape. *Agriculture, Ecosystems & Environment*, *61*(2–3), 145–154. https://doi.org/10.1016/S0167-8809(96)01100-0
73. Schaffer, M., & Wratten, S. D. (1994). Bumblebee (Bombus terrestris) movement in an intensive farm landscape. *Proceedings of the 47th NZ Plant Protection Conference*, 253–256.
74. Schneider, S. S., & Hall, H. G. (1997). Diet selection and foraging distances of African and European-African hybrid honey bee colonies in Costa Rica. *Insectes Sociaux*, *44*(2), 171–187. https://doi.org/10.1007/s000400050039
75. Silva, A. G., Pinto, R. S., Contrera, F. A. L., Albuquerque, P. M. C., & Rêgo, M. M. C. (2014). Foraging distance of *Melipona subnitida* Ducke (Hymenoptera: Apidae). *Sociobiology*, *61*(4), 494–501.
76. Smith, J. P., Heard, T. A., Beekman, M., & Gloag, R. (2017). Flight range of the Australian stingless bee *Tetragonula carbonaria* (Hymenoptera: Apidae). *Austral Entomology*, *56*(1), 50–53.
77. Southwick, E. E., & Buchmann, S. L. (1995). Effects of horizon landmarks on homing success in honey bees. *The American Naturalist*, *146*(5), 748–764. https://doi.org/10.1086/285823
78. Steffan-Dewenter, I., & Kuhn, A. (2003). Honeybee foraging in differentially structured landscapes. *Proceedings of the Royal Society of London. Series B: Biological Sciences*, *270*(1515), 569–575. https://doi.org/10.1098/rspb.2002.2292
79. Tasei, J.-N., Delaude, A., Carré, S., de la Condamine, F., Aletru, J., Nardi, L., & Le Menaheze, J. (1984). Efficacité pollinisatrice de *Megachile rotundata* F.(Hym., Megachilidae) utilisée sur luzerne (*Medicago sativa* L.). *Agronomie*, *4*(7), 653–662.
80. van Nieuwstadt, M. G. L., & Ruano Iraheta, C. E. (1996). Relation between size and foraging range in stingless bees (Apidae, Meliponinae). *Apidologie*, *27*(4), 219–228. https://doi.org/10.1051/apido:19960404
81. Vicens, N., & Bosch, J. (2000). Nest site orientation and relocation of populations of the orchard pollinator *Osmia cornuta* (Hymenoptera: Megachilidae). *Environmental Entomology*, *29*(1), 69–75. https://doi.org/10.1603/0046-225X-29.1.69
82. Visscher, P. K., & Seeley, T. D. (1982). Foraging strategy of honeybee colonies in a temperate deciduous forest. *Ecology*, *63*(6), 1790. https://doi.org/10.2307/1940121

83. Waddington, K. D., Herbert, T. J., Visscher, P. K., & Richter, M. R. (1994). Comparisons of forager distributions from matched honey bee colonies in suburban environments. *Behavioral Ecology and Sociobiology*, *35*(6), 423–429.
84. Walther-Hellwig, K., & Frankl, R. (2000). Foraging habitats and foraging distances of bumblebees, *Bombus* spp. (Hym., Apidae), in an agricultural landscape. *Journal of Applied Entomology*, *124*(7–8), 299–306. https://doi.org/10.1046/j.1439-0418.2000.00484.x
85. Westphal, C., Steffan-Dewenter, I., & Tscharntke, T. (2006). Bumblebees experience landscapes at different spatial scales: Possible implications for coexistence. *Oecologia*, *149*(2), 289–300. https://doi.org/10.1007/s00442-006-0448-6
86. Wille, A. (1976). Las abejas jicotes del género *Melipona* (Apidae: Meliponini) de Costa Rica. *Revista de Biología Tropical*, *24*(1), 123–147.
87. Wille, A., & Orozco, E. (1970). The life cycle and behavior of the social bee *Lasioglossum* (*Dialictus*) *umbripenne* (Hymenoptera: Halictidae). *Revista de Biología Tropical*, *17*(2), 199–245.
88. Wolf, S., & Moritz, R. F. A. (2008). Foraging distance in *Bombus terrestris* L. (Hymenoptera: Apidae). *Apidologie*, *39*(4), 419–427. https://doi.org/10.1051/apido:2008020
89. Wood, T. J., Holland, J. M., Hughes, W. O. H., & Goulson, D. (2015). Targeted agri-environment schemes significantly improve the population size of common farmland bumblebee species. *Molecular Ecology*, *24*(8), 1668–1680. https://doi.org/10.1111/mec.13144
90. Zurbuchen, A., Landert, L., Klaiber, J., Müller, A., Hein, S., & Dorn, S. (2010A). Maximum foraging ranges in solitary bees: Only few individuals have the capability to cover long foraging distances. *Biological Conservation*, *143*(3), 669–676. https://doi.org/10.1016/j.biocon.2009.12.003
91. Zurbuchen, A., Bachofen, C., Müller, A., Hein, S., & Dorn, S. (2010B). Are landscape structures insurmountable barriers for foraging bees? A mark-recapture study with two solitary pollen specialist species. *Apidologie*, *41*(4), 497–508. https://doi.org/10.1051/apido/2009084

Section S2. List of additional publications that provided unclassifiable foraging range measurements.

Measurements from the following publications were not included in analyses as they could not be classified into typical or maximum ranges. These include, for example, studies that only provide an anecdotal account of foraging range or quantified a minimum distance between nesting locations and food patches. These measurements are provided here for general interest. NB: Publications that occur on both lists (i.e., that provided both classifiable and unclassifiable measurements) are emboldened.

1. **Abrol, D. P. (1988). Foraging range of subtropical bees, *Megachile flavipes, Megachile nana* (Hymenoptera: Megachilidae) and *Apis florea* (Hymenoptera: Apidae). *Journal of the Indian Institute of Science*, *68*(1–2), 43.**
2. Bacon, O. G., Burton, V. E., MacSwain, J. W., Marble, V. L., Stanger, W., & Thorp, R. W. (1965). *Pollinating alfalfa with leaf-cutting bees*.
3. Beil, M., Horn, H., & Schwabe, A. (2008). Analysis of pollen loads in a wild bee community (Hymenoptera: Apidae) – a method for elucidating habitat use and foraging distances. *Apidologie*, *39*(4), 456–467. https://doi.org/10.1051/apido:2008021
4. Bennet, D. G., Kelly, D., & Clemens, J. (2018). Food plants and foraging distances for the native bee *Lasioglossum sordidum* in Christchurch Botanic Gardens. *New Zealand Journal of Ecology*, 42(1), 40-47.
5. Bohart, G. E., & Nye, W. P. (1956). Bees. Foraging for nectar and pollen. *Gleanings in Bee Culture*, *84*(10), 602–606.
6. Fabre, J. H. (1914). *The Mason Bees*. New York: Dodd, Mead.
7. Gathmann, A., & Tscharntke, T. (2002). Foraging ranges of solitary bees. *Journal of Animal Ecology*, *71*(5), 757–764. https://doi.org/10.1046/j.1365-2656.2002.00641.x
8. Kapyla, M. (1978). Foraging distance of a small solitary bee, *Chelostoma maxillosum* (Hymenoptera, Megachilidae). *Annnales Etomologici Fennici*, *44*, 63–64.
9. Linsley, E. G., & McSwain, J. W. (1942). The Parasites, predators, and inquiline associates of *Anthophora linsleyi*. *American Midland Naturalist*, *27*(2), 402. https://doi.org/10.2307/2421008
10. Menke, H. (1954). Insect pollination in relation to alfalfa seed production in Washington. *Washington Agricultural Experimental Station Bulletin*, *555*.

11. **Packer, J. S. (1970). *The flight and foraging behavior of the alkali bee (Nomia melanderi) and the alfalfa leaf-cutter bee (Megachile rotundata)*. Utah State University.**
12. Robertson, D. R. (1966). Observations on the alfalfa leaf-cutter bee Megachile rotundata at Hodgson, Manitoba in 1966. *Proc Entomol Soc Manit*, *22*, 34–37.
13. Roubik, D. W. (1992). *Ecology and natural history of tropical bees*. Cambridge University Press.
14. Rust, R. W. (1990). Spatial and temporal heterogeneity of pollen foraging in *Osmia lignaria propinqua* (Hymenoptera: Megachilidae). *Environmental Entomology*, *19*(2), 332–338. https://doi.org/10.1093/ee/19.2.332
15. Schmid-Egger, C., Risch, R., & Niehuis, O. (1995). Die wildbienen und wespen in Rheinland-Pfalz. *Fauna Und Flora in Rheinland-Pfalz, Beiheft*, *16*, 1–296.
16. Tepedino, V. J. (1983). An open-field test of *Megachile Rotundata* as a potential pollinator in hybrid carrot seed fields. *Journal of Apicultural Research*, *22*(1), 64–68. https://doi.org/10.1080/00218839.1983.11100561
17. Vansell, G. H., & Todd, F. E. (1946). Alfalfa tripping by insects. *American Society of Agronomy Journal.*, *38*, 470–488.
18. **Wille, A. (1976). Las abejas jicotes del género *Melipona* (Apidae: Meliponini) de Costa Rica. *Revista de Biología Tropical*, *24*(1), 123–147.**

Section S3. Literature sources of intertegular distance (ITD) measurements and sociality used within this study

ITD measurements

1. Bommarco, R., Biesmeijer, J. C., Meyer, B., Potts, S. G., Pöyry, J., Roberts, S. P. M., Steffan-Dewenter, I., & Öckinger, E. (2010). Dispersal capacity and diet breadth modify the response of wild bees to habitat loss. *Proceedings of the Royal Society B: Biological Sciences*, *277*(1690), 2075–2082. https://doi.org/10.1098/rspb.2009.2221
2. Borges, R. C., Padovani, K., Imperatriz-Fonseca, V. L., & Giannini, T. C. (2020). A dataset of multi-functional ecological traits of Brazilian bees. *Scientific Data*, *7*(1), 1–9.
3. Castillo, R. C. del, & Fairbairn, D. J. (2012). Macroevolutionary patterns of bumblebee body size: Detecting the interplay between natural and sexual selection. *Ecology and Evolution*, *2*(1), 46–57. https://doi.org/10.1002/ece3.65
4. Fortel, L., Henry, M., Guilbaud, L., Guirao, A. L., Kuhlmann, M., Mouret, H., Rollin, O., & Vaissière, B. E. (2014). Decreasing abundance, increasing diversity and changing structure of the wild bee community (Hymenoptera: Anthophila) along an urbanization gradient. *PLOS ONE*, *9*(8), e104679. https://doi.org/10.1371/journal.pone.0104679
5. Fowler, R. E. (2014). *An investigation into bee assemblage change along an urban-rural gradient.* PhD thesis. University of Birmingham.
6. Kendall, L. K., Rader, R., Gagic, V., Cariveau, D. P., Albrecht, M., Baldock, K. C. R., Freitas, B. M., Hall, M., Holzschuh, A., Molina, F. P., Morten, J. M., Pereira, J. S., Portman, Z. M., Roberts, S. P. M., Rodriguez, J., Russo, L., Sutter, L., Vereecken, N. J., & Bartomeus, I. (2019). Pollinator size and its consequences: Robust estimates of body size in pollinating insects. *Ecology and Evolution*, *9*(4), 1702–1714. https://doi.org/10.1002/ece3.4835
7. Mayes, D. M. (2019). *Wild bee responses to land use change: Investigating the role of bee body size.* PhD thesis. University of Kansas.
8. Polidori, C., Jorge, A., & Ornosa, C. (2020). Antennal morphology and sensillar equipment vary with pollen diet specialization in Andrena bees. *Arthropod Structure & Development*, *57*, 100950. https://doi.org/10.1016/j.asd.2020.100950
9. Quezada-Euán, J. J. G., Sanabria-Urbán, S., Smith, C., & Cueva del Castillo, R. (2019). Patterns of sexual size dimorphism in stingless bees: Testing Rensch's rule and potential causes in highly eusocial bees (Hymenoptera: Apidae, Meliponini). *Ecology and Evolution*, *9*(5), 2688–2698.
10. Redhead, J. W., Dreier, S., Bourke, A. F. G., Heard, M. S., Jordan, W. C., Sumner, S., Wang, J., & Carvell, C. (2016). Effects of habitat composition and landscape structure on worker foraging distances of five bumble bee species. *Ecological Applications*, *26*(3), 726–739. https://doi.org/10.1890/15-0546

Sociality

1. Davison, P. J., & Field, J. (2016). Social polymorphism in the sweat bee *Lasioglossum* (*Evylaeus*) *calceatum*. *Insectes Sociaux*, *63*(2), 327–338.
2. Paxton, R. J., Ayasse, M., Field, J., & Soro, A. (2002). Complex sociogenetic organization and reproductive skew in a primitively eusocial sweat bee, *Lasioglossum malachurum*, as revealed by microsatellites. *Molecular Ecology*, *11*(11), 2405–2416.
3. Pesenko, Y., Banaszak, J., Radchenko, V., & Cierzniak, T. (2000). *Bees of the family Halictidae (excluding Sphecodes) of Poland: Taxonomy, ecology, bionomics*.
4. Vickruck, J. L., & Richards, M. H. (2018). Linear dominance hierarchies and conditional reproductive strategies in a facultatively social carpenter bee. *Insectes Sociaux*, *65*(4), 619–629.
5. Wille, A., & Orozco, E. (1970). The life cycle and behavior of the social bee *Lasioglossum* (*Dialictus*) *umbripenne* (Hymenoptera: Halictidae). *Revista de Biología Tropical*, *17*(2), 199–245.
