# Supplementary material for: The potential and realized foraging movements of bees are differentially determined by body size and sociality
Source: Ecology. 2022 Sep 1;103(11):e3809. doi: 10.1002/ecy.3809 (PMC9786665; doi:10.1002/ecy.3809)
Supplement: Supplementary file 2 — Appendix S2 [file ECY-103-e3809-s001.pdf]

Authors: Liam K. Kendall, John M. Mola, Zachary M. Portman, Daniel P. Cariveau, Henrik G. Smith, Ignasi Bartomeus
Title: The potential and realized foraging movements of bees are differentially determined by body size and sociality
Journal: Ecology

Appendix S2. Additional summary information and figures, regarding, i) sample sizes from publications that provided foraging range measurements (Figure S1), and ii) bee foraging ranges at the genus-level* (except for *Apis mellifera* and *Bombus terrestris)* (Figure S2).

Publication sample sizes

The median sample sizes of individuals (or colonies) used to measure foraging ranges were between 93 – 228 in studies of highly eusocial species, 65 – 105 in studies of primitively eusocial species, and between 30 – 39 in studies of solitary species (Figure S1). Missingness in sample sizes (i.e., studies that did not report actual sample sizes or provide enough information to calculate it), ranged from 33% – 72% for studies of highly eusocial species, 10% – 40% for studies of primitively eusocial species, and 38% – 57% for studies of solitary species. Studies with low sample sizes were typically bee tracking, mark-recapture, or molecular studies. The largest sample size was from a waggle dance study, in which 4562 dances were decoded (Couvillon et al. 2015).

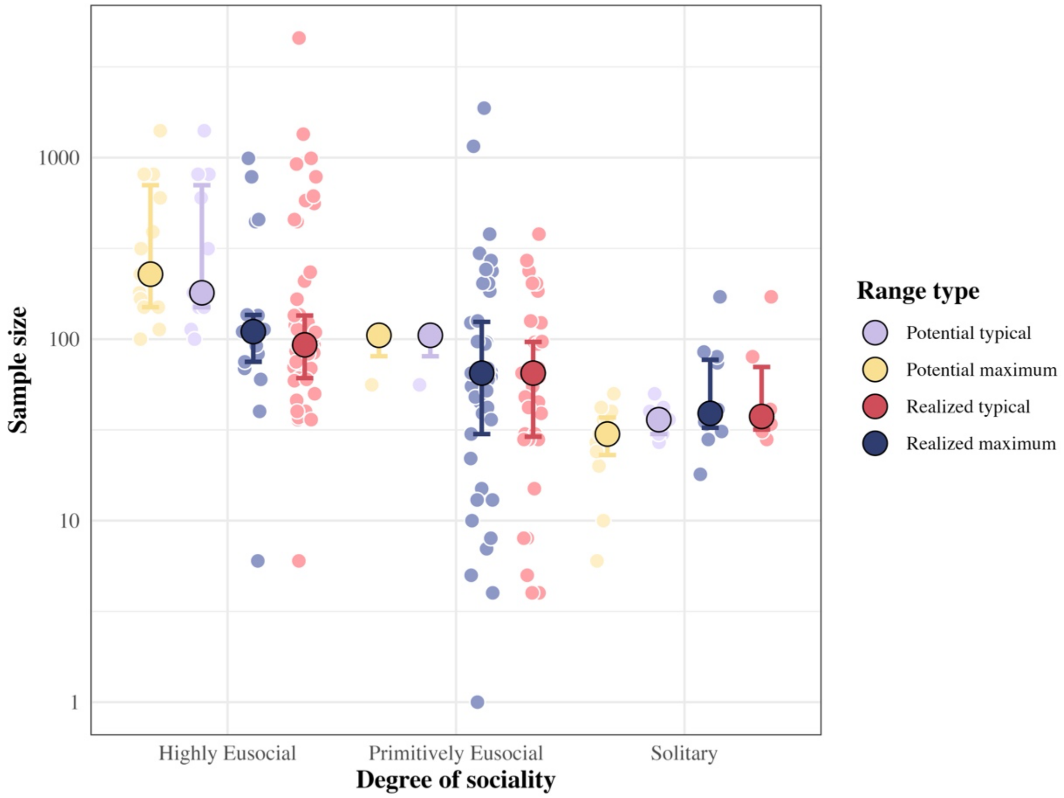

Figure S1. Publication sample sizes for different foraging range types and bee social groups. Large dots ± error bars indicate medians and interquartile ranges. Background circles denote raw data. Y-axis is shown on the log-10 scale.

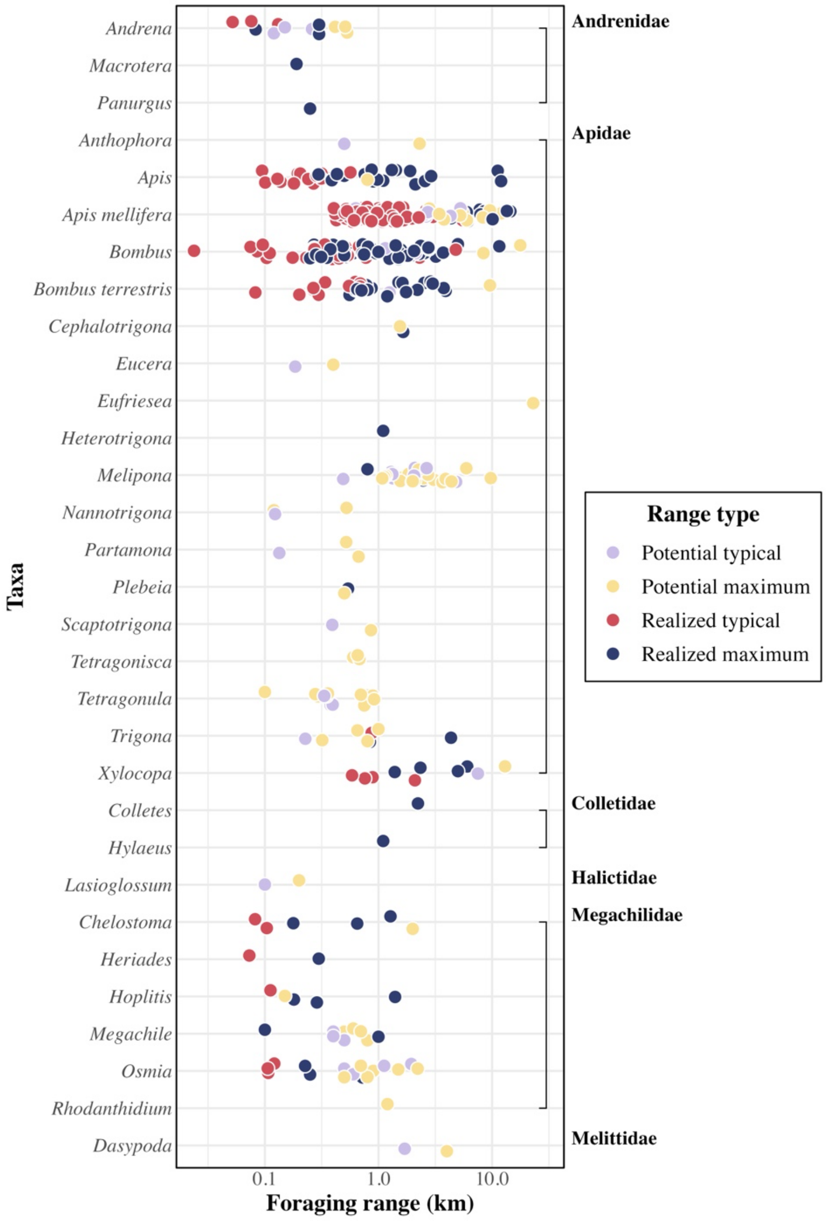

Figure S2. Bee foraging range measurements compiled during the literature review. Measurements are collated at the genera-level except for *Apis mellifera* and *Bombus terrestris,* which are shown separately due to the large sample sizes for each species. X-axis is shown on the log-10 scale.

References

Couvillon, M. J., F. C. Riddell Pearce, C. Accleton, K. A. Fensome, S. K. L. Quah, E. L. Taylor, and F. L. W. Ratnieks. "Honey Bee Foraging Distance Depends on Month and Forage Type." *Apidologie* 46, no. 1 (2015): 61–70. https://doi.org/10.1007/s13592-014-0302-5.
