# Supplementary material for: The potential and realized foraging movements of bees are differentially determined by body size and sociality
Source: Ecology. 2022 Sep 1;103(11):e3809. doi: 10.1002/ecy.3809 (PMC9786665; doi:10.1002/ecy.3809)
Supplement: Supplementary file 3 — Appendix S3 [file ECY-103-e3809-s003.pdf]

Authors: Liam K. Kendall, John M. Mola, Zachary M. Portman, Daniel P. Cariveau, Henrik G. Smith, Ignasi Bartomeus
Title: The potential and realized foraging movements of bees are differentially determined by body size and sociality
Journal: Ecology

Appendix S3. Bee foraging range model predictive performance and accuracy

To demonstrate and test the predictive performance of our most-predictive model (see results), we explore how model predictions differ depending on the random structure (which can be chosen by users of the *R* function `foraging.range` within the `pollimetry` package (Kendall et al. 2019):

i) ~ 1|phylogeny + 1|species

ii) ~ 1|phylogeny

iii) fixed effects only (random effects set to zero)

These three scenarios correspond to predictions of species present in the original database, species not present but for which you can leverage the information encoded in their phylogeny, and completely unrepresented species respectively.

We then compared these formulations with pre-existing models of foraging range by calculating the mean absolute error (MAE) between predicted values and actual values in our dataset. Specifically, we compared our model against predicted estimates from Greenleaf et al. (2007)'s maximum and typical homing range models. We calculated the MAE independently for potential and realized measurements. We acknowledge the bias introduced from assessing predictive accuracy between actual and predicted values, where all actual values were used in model creation. However, the aim of this exercise is to compare the performance among

models, and show their usability, and not to test their true (out-of-sample) predictive power. Furthermore, the complex data structure (91 publications, seven measurement types, 81 species) made splitting the dataset into training and testing sets unrepresentative. Note that Greenleaf et al. (2007) models were parameterized using a subset of the total dataset used herein, so exhibit some of the same bias.

Predictive performance and accuracy

Across all types of foraging range, we found that the model incorporating measurement type and sociality had considerably greater predictive accuracy relative to pre-existing models. We found that improved predictive accuracy was most notable for realized estimates (Figure S1). The mean absolute error of potential estimates was reduced by up to 15% for typical range (130 m) and, up to 29% for maximum foraging range (488 m). For realized estimates, predictive error was reduced by 65% – 74% for typical range (910 m – 1.04 km) and reduced by 69% – 76% for maximum range (3.35 km – 3.68 km). Predictions for all species in our dataset, based on each of the three model formulations, are provided in Figure S2.

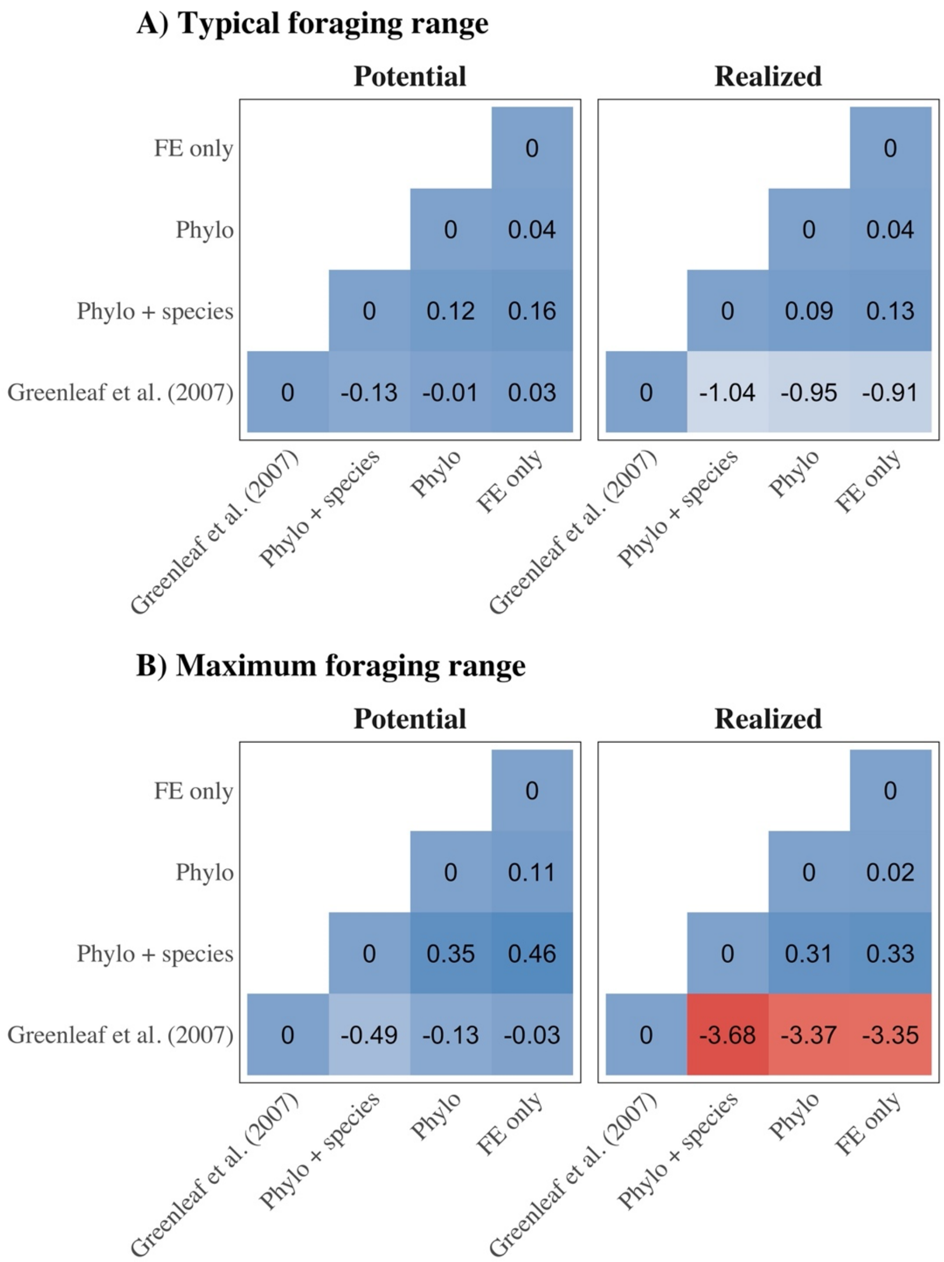

Figure S1. Pairwise comparison of marginal absolute error (Δ MAE) in kilometers for typical and maximum foraging ranges of bees (potential and realized) between new foraging range models and pre-existing models (Greenleaf et al. 2007). Blue values denote marginal or null differences between models in terms of precision, whereas white and red values indicate lower model error in models in the columns relative to the rows. Predictions come from either Greenleaf et al. (2007)'s homing range model for maximum or typical foraging range, or our most-predictive model: ~ body mass * sociality * measurement type, with differing random effect structures: Phylo + species: ~1|phylogeny + 1|species, Phylo: ~1|phylogeny, and FE only: fixed effects only (random effects set to zero).

A demonstrative example

To demonstrate the use of the developed models, we used a bee of the same size (body mass: 23 mg) from each social group, such as *Apis mellifera* (highly eusocial), a small *Bombus bifarius* worker (primitively eusocial) and *Osmia cornifrons* (solitary), to generate predictions of typical and maximum foraging ranges, using three different model parameterizations (Figure S3).

Model predictions from each formulation were highly similar for primitively eusocial and solitary species, irrespective of the random effect structure. For highly eusocial species, predictions that considered phylogeny, and especially both phylogeny and the species effect for *Apis mellifera*, increased both the median estimate of foraging range by as much as 123% (3.14 km) and its associated uncertainty (by as much as 7.24 km). Potential typical foraging ranges were, on average, 47%, 53%, and 66% larger than realized ranges for highly eusocial, solitary and primitively eusocial bee species, respectively. In contrast, potential maximum foraging ranges were, on average, 65% and 36% larger than realized ranges for the primitively eusocial bee and solitary species, and 29% shorter for the highly eusocial species.

Foraging ranges for the highly eusocial species were considerably larger than the ranges of primitively eusocial and solitary species. Maximum foraging ranges (potential and realized) of the highly eusocial species were between 23% – 147% and 356 % – 800% larger than the primitively eusocial species, and between 96% – 343% and 294% – 793% larger than the solitary species. Maximum foraging ranges of the primitively eusocial species were between 59% – 80% larger (potential) and between 1% – 14% shorter (realized) than the solitary species.

Typical foraging ranges (potential and realized) of the highly eusocial species were between 116% – 328% and 241 % – 573% larger than the primitively eusocial species, and between 58% – 262% and 82% – 305% larger than the solitary species. Typical foraging ranges of the primitively eusocial species were between 15% – 26% (potential) and 39% – 47% shorter (realized) than the solitary species.

References

Greenleaf, S. S., N. M. Williams, R. Winfree, and C. Kremen. "Bee Foraging Ranges and Their Relationship to Body Size." *Oecologia* 153, no. 3 (2007): 589–96.

Kendall, L. K., R. Rader, V. Gagic, D. P. Cariveau, M. Albrecht, K. C. R. Baldock, B. M. Freitas, et al. "Pollinator Size and Its Consequences: Robust Estimates of Body Size in Pollinating Insects." *Ecology and Evolution* 9, no. 4 (2019): 1702–14.

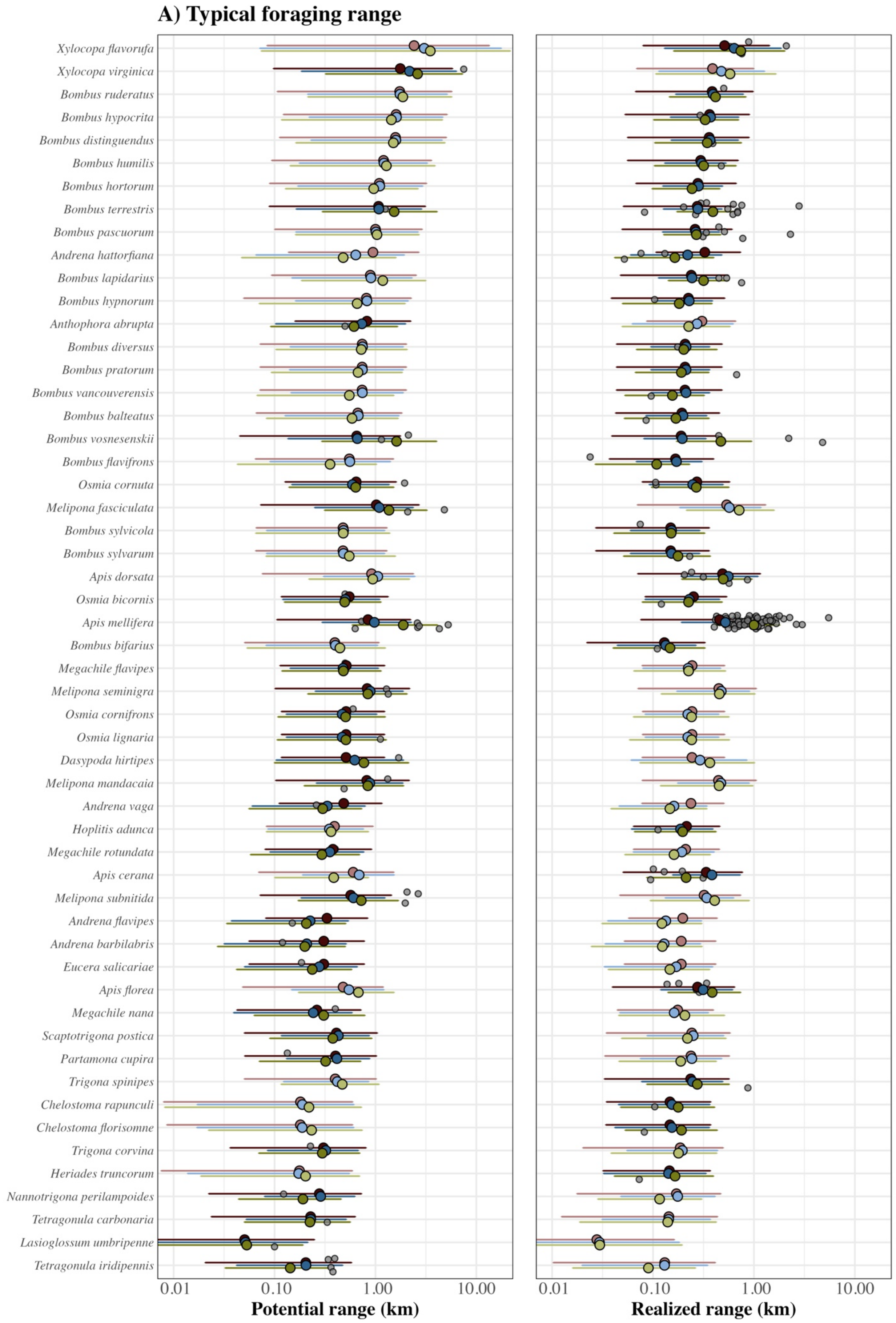
A) Typical foraging range
Xylocopa flavorufa
Xylocopa virginica
Bombus ruderatus
Bombus hypocrita
Bombus distinguendus
Bombus humilis
Bombus hortorum
Bombus terrestris
Bombus pascuorum
Andrena hattorfiana
Bombus lapidarius
Bombus hypnorum
Anthophora abrupta
Bombus diversus
Bombus pratorum
Bombus vancouverensis
Bombus balteatus
Bombus vosnesenskii
Bombus flavifrons
Osmia cornuta
Melipona fasciculata
Bombus sylvicola
Bombus sylvarum
Apis dorsata
Osmia bicornis
Apis mellifera
Bombus bifarius
Megachile flavipes
Melipona seminigra
Osmia cornifrons
Osmia lignaria
Dasypoda hirtipes
Melipona mandacaia
Andrena vaga
Hoplitis adunca
Megachile rotundata
Apis cerana
Melipona subnitida
Andrena flavipes
Andrena barbilabris
Eucera salicariae
Apis florea
Megachile nana
Scaptotrigona postica
Partamona cupira
Trigona spinipes
Chelostoma rapunculi
Chelostoma florisomne
Trigona corvina
Heriades truncorum
Nannotrigona perilampoides
Tetragonula carbonaria
Lasioglossum umbripenne
Tetragonula iridipennis
0.01
0.10
1.00
10.00
Potential range (km)
0.01
0.10
1.00
10.00
Realized range (km)

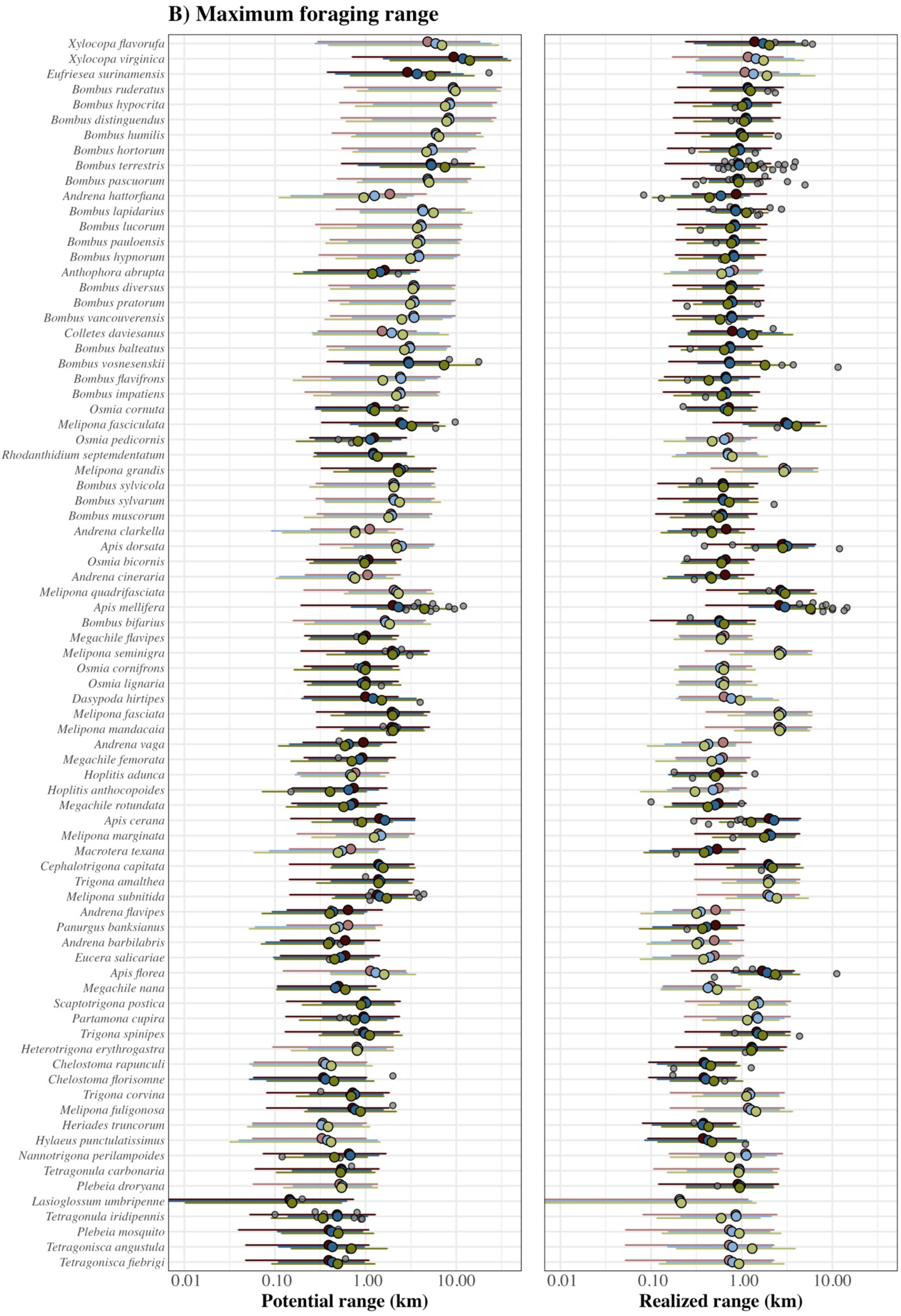

B) Maximum foraging range
Xylocopa flavorufa
Xylocopa virginica
Eufriesea surinamensis
Bombus ruderatus
Bombus hypocrita
Bombus distinguendus
Bombus humilis
Bombus hortorum
Bombus terrestris
Bombus pascuorum
Andrena hattorfiana
Bombus lapidarius
Bombus lucorum
Bombus pauloensis
Bombus hypnorum
Anthophora abrupta
Bombus diversus
Bombus pratorum
Bombus vancouverensis
Colletes daviesanus
Bombus balteatus
Bombus vosnesenskii
Bombus flavifrons
Bombus impatiens
Osmia cornuta
Melipona fasciculata
Osmia pedicornis
Rhodanthidium septemdentatum
Melipona grandis
Bombus sylvicola
Bombus sylvarum
Bombus muscorum
Andrena clarkella
Apis dorsata
Osmia bicornis
Andrena cineraria
Melipona quadrifasciata
Apis mellifera
Bombus bifarius
Megachile flavipes
Melipona seminigra
Osmia cornifrons
Osmia lignaria
Dasypoda hirtipes
Melipona fasciata
Melipona mandacaia
Andrena vaga
Megachile femorata
Hoplitis adunca
Hoplitis anthocopoides
Megachile rotundata
Apis cerana
Melipona marginata
Macrotera texana
Cephalotrigona capitata
Trigona amalthea
Melipona subnitida
Andrena flavipes
Panurgus banksianus
Andrena barbilabris
Eucera salicariae
Apis florea
Megachile nana
Scaptotrigona postica
Partamona cupira
Trigona spinipes
Heterotrigona erythrogastra
Chelostoma rapunculi
Chelostoma florisomne
Trigona corvina
Melipona fuliginosa
Heriades truncorum
Hylaeus punctulatissimus
Nannotrigona perilampoides
Tetragonula carbonaria
Plebeia droryana
Lasioglossum umbripenne
Tetragonula iridipennis
Plebeia mosquito
Tetragonisca angustula
Tetragonisca fiebrigi
0.01
0.10
1.00
10.00
Potential range (km)
0.01
0.10
1.00
10.00
Realized range (km)

Figure S2A-B. Typical **(A)** and maximum **(B)** foraging range estimates for potential and realized ranges of bees. Species are sorted by body size (body mass, mg) in descending order from largest to smallest. Points (red, blue, or green) indicate posterior mean estimates ± 95% credible intervals from the three model formulations introduced in the demonstrative example section. Dark-colored points denote species that had an estimate within the dataset, whereas light-colored points denote species which did not have data in the dataset but did have a datapoint in any group (i.e., potential or realized) or foraging range type (typical or maximum). X axes are shown on the log-10 scale.

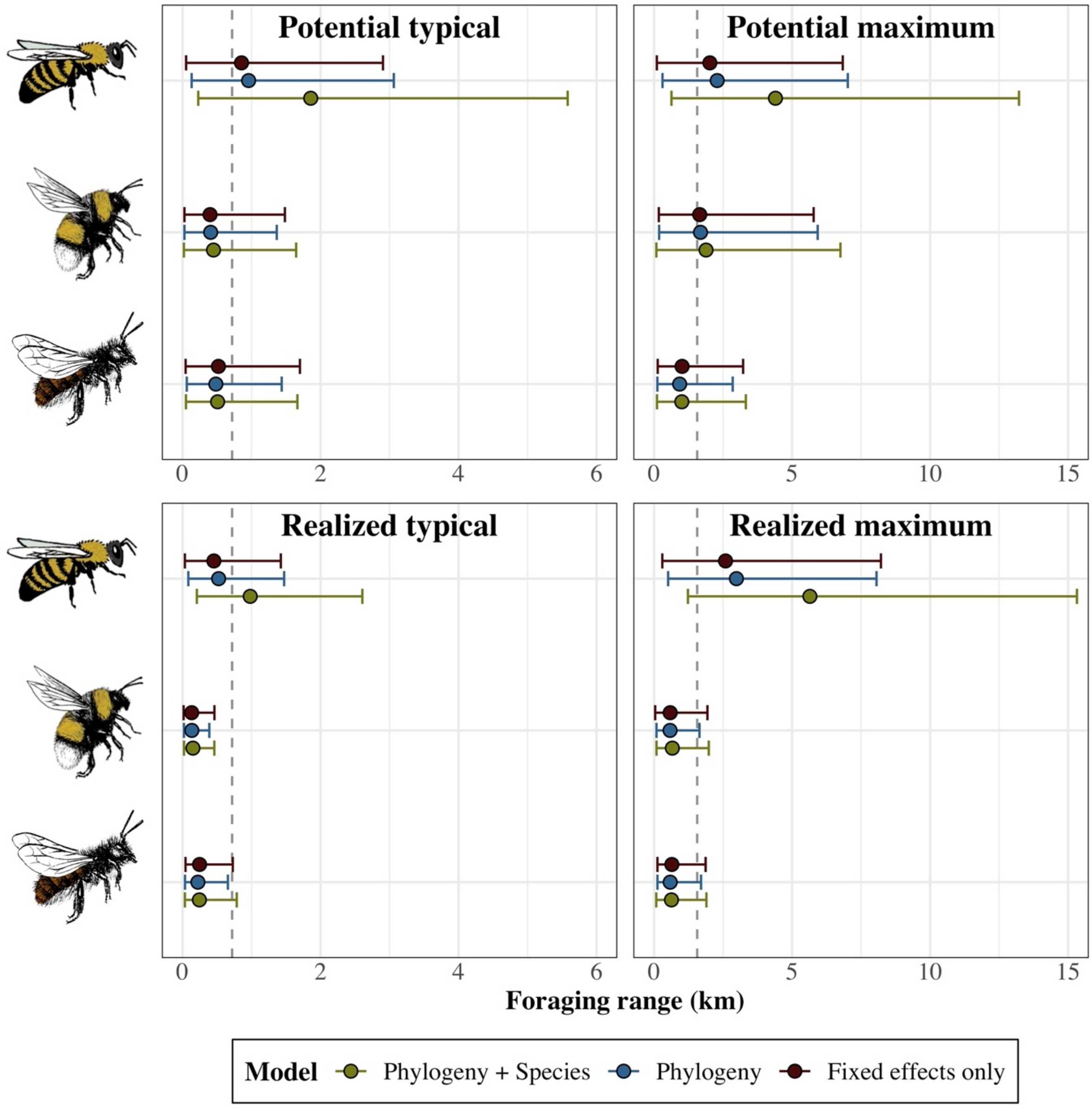

Figure S3. Posterior predictive distributions of potential and realized foraging range for a highly eusocial bee **(top)**, primitively eusocial bee **(middle)**, and a solitary bee **(bottom)**, with the same body size (dry body mass: 23 mg). We generated predictions using three different model parameterizations, which varied in their random effect structure (see legend). Estimates from Greenleaf et al. (2007)'s homing range models are provided as vertical dashed lines in the background. Dots and error bars indicate median values ± 95% credible intervals. Bee drawings, courtesy of Johanna Yourstone, used with permission.

Any use of trade, firm, or product names is for descriptive purposes only and does not imply endorsement by the U.S. Government.
